# Evidence for Trait Related Theory of Mind Impairment in First Episode Psychosis Patients and Its Relationship with Processing Speed: A 3 Year Follow-up Study

**DOI:** 10.3389/fpsyg.2016.00592

**Published:** 2016-05-04

**Authors:** Rosa Ayesa-Arriola, Esther Setién-Suero, Karl D. Neergaard, Adele Ferro, Mar Fatjó-Vilas, Marcos Ríos-Lago, Soraya Otero, Jose M. Rodríguez-Sánchez, Benedicto Crespo-Facorro

**Affiliations:** ^1^Department of Psychiatry, Marqués de Valdecilla University Hospital, IDIVAL, School of Medicine, University of CantabriaSantander, Spain; ^2^Centro Investigación Biomédica en Red de Salud Mental (CIBERSAM)Madrid, Spain; ^3^Department of Chinese and Bilingual Studies, The Hong Kong Polytechnic UniversityHong Kong, China; ^4^Department of Experimental Clinical Medicine, University of UdineUdine, Italy; ^5^Department of Mental Health and Neuroscience, Fondazione IRCCCS, Granda Ospedale Maggiore PoliclinicoMilan, Italy; ^6^Departament of Animal Biology, Faculty of Biology, Institute of Biomedicine of the University of Barcelona, Universitat de BarcelonaBarcelona, Spain; ^7^Department de Psicologia Basica II, Faculty of Psychology, Universidad de Educación a DistanciaMadrid, Spain; ^8^Unidad de Daño Cerebral, Red Menni de Atención al Daño Cerebral, Hospital Beata María AnaMadrid, Spain; ^9^Child and Adolescent Psychiatry Unit, Marqués de Valdecilla University HospitalSantander, Spain; ^10^Centro Investigación en Red de Salud Mental de Bizkaia-OsakidetzaBiscay, Spain

**Keywords:** first episode psychosis, theory of mind, processing speed, clinical symptoms, schizophrenia

## Abstract

This study aimed to confirm whether first-episode psychosis patients present a stable trait impairment in theory of mind (ToM) and to examine the potential relationship between ToM and clinical symptomatology and neurocognition. Patients with a first episode of psychosis (*N* = 160) and healthy controls (*N* = 159) were assessed with an extensive neuropsychological test battery, which included a mental state decoding task known as “The Reading the Mind in the Eyes” (Eyes test), at baseline and reassessed after 1 and 3 years. The clinical group performed below healthy controls on the Eyes test while not showing test-retest differences between baseline and follow-up administrations. Analyses revealed age, education and premorbid IQ as potential moderators. Poorer performance on the Eyes test was not linked to clinical symptomatology but was associated with greater neurocognitive deficit, particularly related to processing speed. The persistence of ToM deficits in patients suggests that there are trait related metalizing impairments in first episode psychosis. This study shows the influence of processing speed and moderator variables on efficient ToM.

## Introduction

There has been a growing interest over the last three decades in theory of mind (ToM), defined as the ability to attribute mental states to oneself and others (Baron-Cohen, 1991). ToM impairment has been confirmed in schizophrenia (Sprong et al., 2007; Bora et al., 2009), with important implications for social functioning (Bora et al., 2006). However, whether ToM deficits are state dependent or trait characteristics, and the nature of the relationship with neurocognitive deficits and clinical symptoms remains controversial.

A review of the literature supports the hypothesis that deficits in ToM are specific rather than secondary to symptoms or general cognitive decline. Bora et al. remark in their meta-analysis (Bora et al., 2009), that the persistence of ToM deficits, even in “remitted” patients, suggests trait related metalizing impairments in schizophrenia. However, a more severe ToM impairment related to positive and negative symptoms (Bora et al., 2008), and well as disorganization and other behavioral symptoms (Brüne, 2005b), point toward specific ToM deficits in schizophrenia as state dependent (Pousa et al., 2008). In terms of neurocognitive functioning, studies are contradictory when establishing ToM deficits as an independent trait (Bozikas et al., 2011) or secondary to general neurocognitive dysfunction (Pentaraki et al., 2012), and particularly related to working memory, attention and executive functions (Bozikas et al., 2011).

Regretfully, as So et al. (2010) pointed out, most of the available studies are limited in their generalizability in that they have small samples sizes without appropriate healthy control groups, and few are longitudinal or differ in the measurement of ToM performance. Meanwhile, few studies have examined ToM in first episode psychosis (FEP) patients (Inoue et al., 2006; Thompson et al., 2012; Bora and Pantelis, 2013; Fernandez-Gonzalo et al., 2014). To the best of our knowledge, two studies with FEP presented longitudinal, 1-year follow-up, designs: Addington et al. (2006) with 50 FEP and 55 non-psychiatric controls; and Horan et al. (2012) with 55 FEP patients, but without long-term information for a control group. While studies that investigate ToM impairment in early phases are important to understanding the nature of ToM dysfunction in schizophrenia, longitudinal studies are necessary to investigate the trajectory of ToM deficits and to shed light on the controversy of whether such deficits are state-like or trait-like.

## Aims of the study

The aims of our study were to explore whether there is a difference in ToM performance in FEP patients relative to healthy individuals and to determine the stability on performance at 1 and 3-year follow-ups. We hypothesized that FEP patients would have persistently lower performance compared to that of healthy volunteers. We also aimed to explore the ties between ToM and other neuropsychological and clinical variables. We hypothesized that ToM deficits would be related to neurocognitive decline and residual symptomatology.

## Methods

### Participants

The study sample comes from a large epidemiological program on first-episode psychosis (PAFIP) at University Hospital Marques de Valdecilla (Santander, Spain). Ethical approval was obtained from the local Ethics Committee. A more detailed description of PAFIP has been previously given (Pelayo-Terán et al., 2008).

The patient group consisted of 160-medication naïve subjects (Age: 16–60, M: 32.11) included in the first-episode psychosis program of Cantabria, Spain, (PAFIP) recruited from January 2005 to December 2010. Written informed consent was obtained from all participants after complete description of the study. The patients met the following criteria: (1) 15–60 years of age; (2) living in the catchment area; (3) were experiencing their first episode of psychosis; (4) had no prior treatment with antipsychotic medication or, if previously treated, a total life-time of adequate antipsychotic treatment of less than 6 weeks; and (5) met the DSM-IV criteria for brief psychotic disorder, schizophreniform disorder, schizophrenia or not otherwise specified (NOS) psychosis. The diagnoses were confirmed through the use of the Structured Clinical Interview for DSM-IV (SCID–I) (First et al., 1996) conducted by an experienced psychiatrist 6 months on from the baseline visit. Diagnoses of FEP patients were classified as: schizophrenia (*N* = 87), schizophreniform disorder (*N* = 40), brief psychotic disorder (*N* = 22) and psychosis NOS (*N* = 11). These patients were randomly assigned to: aripiprazol (*N* = 64), quetiapine (*N* = 46) or ziprasidone (*N* = 50).

A group of 159 healthy volunteers (Age: 15–51, M: 29.01) were initially recruited from the community through advertisements. They had no current or past history of psychiatric, neurological or general medical illnesses, including substance abuse and significant loss of consciousness as determined by using an abbreviated version of the Comprehensive Assessment of Symptoms and History (CASH) (Andreasen et al., 1992).

### Sociodemographic and clinical variables

The patients were screened for demographic and clinical characteristics: age, education and symptoms of psychosis, assessed by mean scores on the Scale for the Assessment of Negative Symptoms (SANS) (Andreasen, 1983) and the Scale for the Assessment of Positive Symptoms (SAPS) (Andreasen, 1984). The SANS and SAPS scores were used in generating dimensions of positive (scores for hallucinations and delusions), disorganized (scores for formal thought disorder, bizarre behavior, and inappropriate affect) and negative (scores for alogia, affective fattening, apathy, and anhedonia) symptoms (Grube et al., 1998). Depressive symptoms were evaluated using the Calgary Depression Scale for Schizophrenia (CDSS) (Addington et al., 1992).

### Neuropsychological assessment

The neuropsychological evaluation was performed at any time between week-6 and week-13, as this time is considered optimal for patients' stabilization (González-Blanch et al., 2007). Trained neuropsychologists administered the tests.

The tests were grouped in the following cognitive domains consistently shown to be impaired in schizophrenia (Nuechterlein et al., 2004): 1- Verbal memory: the Rey Auditory Verbal Learning Test (RAVLT) (Rey, 1964) (list recall score); 2- Visual memory: Rey Complex Figure (RCF) (Osterrieth, 1944) (delayed recall); 3- Working memory: WAIS-III digits forward and backward subtests (Wechler, 1997) (standard total score); 4- Executive function: Trail Making Test (TMT) (Reitan and Wolfson, 1985; Periáñez et al., 2007) (trail B-A score); 5-Processing speed: WAIS-III digit symbol subtest (Wechler, 1997) (standard total score); 6- Motor dexterity: Grooved Pegboard Test (Lezak, 1995) (time to complete with dominant hand); 7- Attention: Continuous Performance Test (CPT) (Cegalis and Bowlin, 1991) (correct responses); 8- Premorbid IQ: WAIS-III vocabulary subtest (Wechler, 1997).

### Theory of mind task

The Reading the Mind in the Eyes (Eyes test) (Baron-Cohen et al., 2001) was applied to assess ToM abilities. The test requires participants to identify the mental state just from the eye region. Studies that have used the Eyes test have provided evidence of a different aspect of ToM ability: mental state decoding skill (Bora et al., 2006).

### Statistical analysis

The Statistical Package for Social Science, version 19.0 (SPSS Inc., Chicago, IL), was used for statistical analyses.

Parametric χ^2^ and *t*-tests were first used to compare patients and healthy volunteers on demographic characteristics and ToM measure. Due to the longitudinal nature of the present study, repeated measure analysis of variance (ANOVA-r), adjusted for the covariates sex, age, education, and premorbid IQ, were then used to compare main effects of group, as well as group-by-time interactions on ToM. The relationship between ToM, and clinical and neuropsychological variables were then tested with Pearson correlations. *Post-hoc* comparisons were Bonferroni corrected. Finally, *t*-test and logistic regression analyses were conducted to overcome correlational limitations. The performance of the regression model was examined via Nagelkerke's R^2^, a measure of the proportion of explained variation in the logistic regression models, and in addition, logistic regression yields odds ratios (ORs), which measures the strengths of associations.

All statistical tests were two-tailed, and significance was determined at the 0.05 level.

## Results

### Sociodemographic and ToM group comparisons

**Figure 1:**
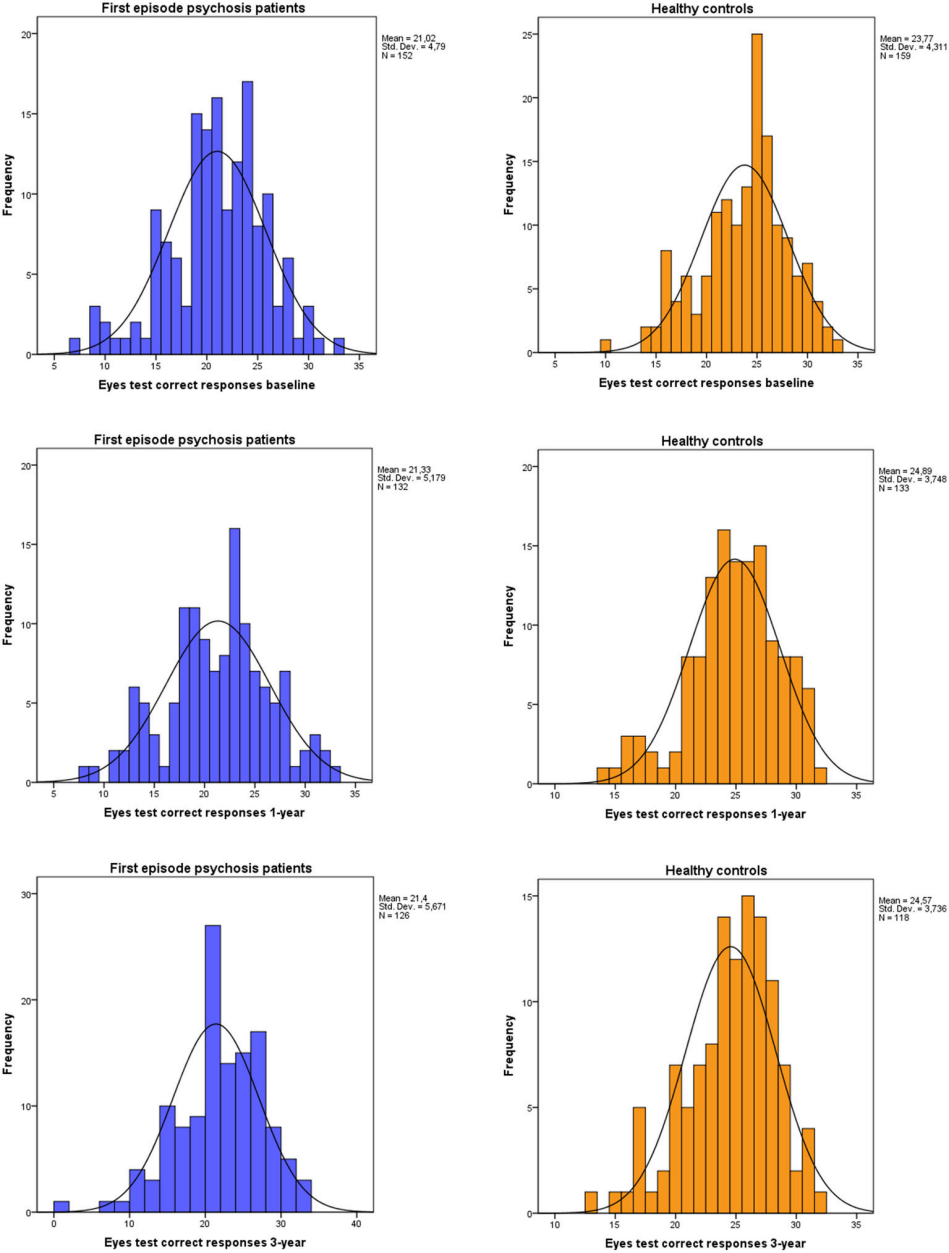
**Distributions of correct responses to the Eyes test at baseline, 1- and 3-year follow-up for both first episode psychosis patients, and healthy controls**.

A comparison of the 160 FEP patients and the 159 healthy controls regarding sociodemographic and ToM characteristics is presented in Table 1. Patients showed significantly reduced skills in ToM as can be seen in Figure 1.

**Table 1:** **Comparison of sociodemographic characteristics and ToM measure for FEP patients and healthy controls**.

	***n***	**FEP patients**	**Healthy controls**	**Statistic**	***p***
		**Mean (*SD*)**	**Mean (*SD*)**		
Sex (female%)	160/159	46.3	39	χ^2^ = 1.72	0.19
Age (years)	160/159	32.17 (10.78)	29.13 (7.93)	*t* = 2.86	0.004^*^
Education (years)	159/159	10.38 (3.55)	10.76 (2.77)	*t* = −1.07	0.284
Premorbid IQ	159/159	9.09 (2.55)	9.54 (2.03)	*t* = −1.73	0.085
Eyes test (baseline)	152/159	21.03 (4.78)	23.77 (4.31)	*t* = −5.34	< 0.001^*^
Eyes test (1-year)	133/133	21.31 (5.18)	24.89 (3.75)	*t* = −6.48	< 0.001^*^
Eyes test (3-year)	124/118	21.33 (5.68)	24.57 (3.74)	*t* = −5.26	< 0.001^*^

Premorbid IQ, WAIS III Vocabulary standard score; Eyes test, correct responses.

* p < 0.05.

**Figure 2:**
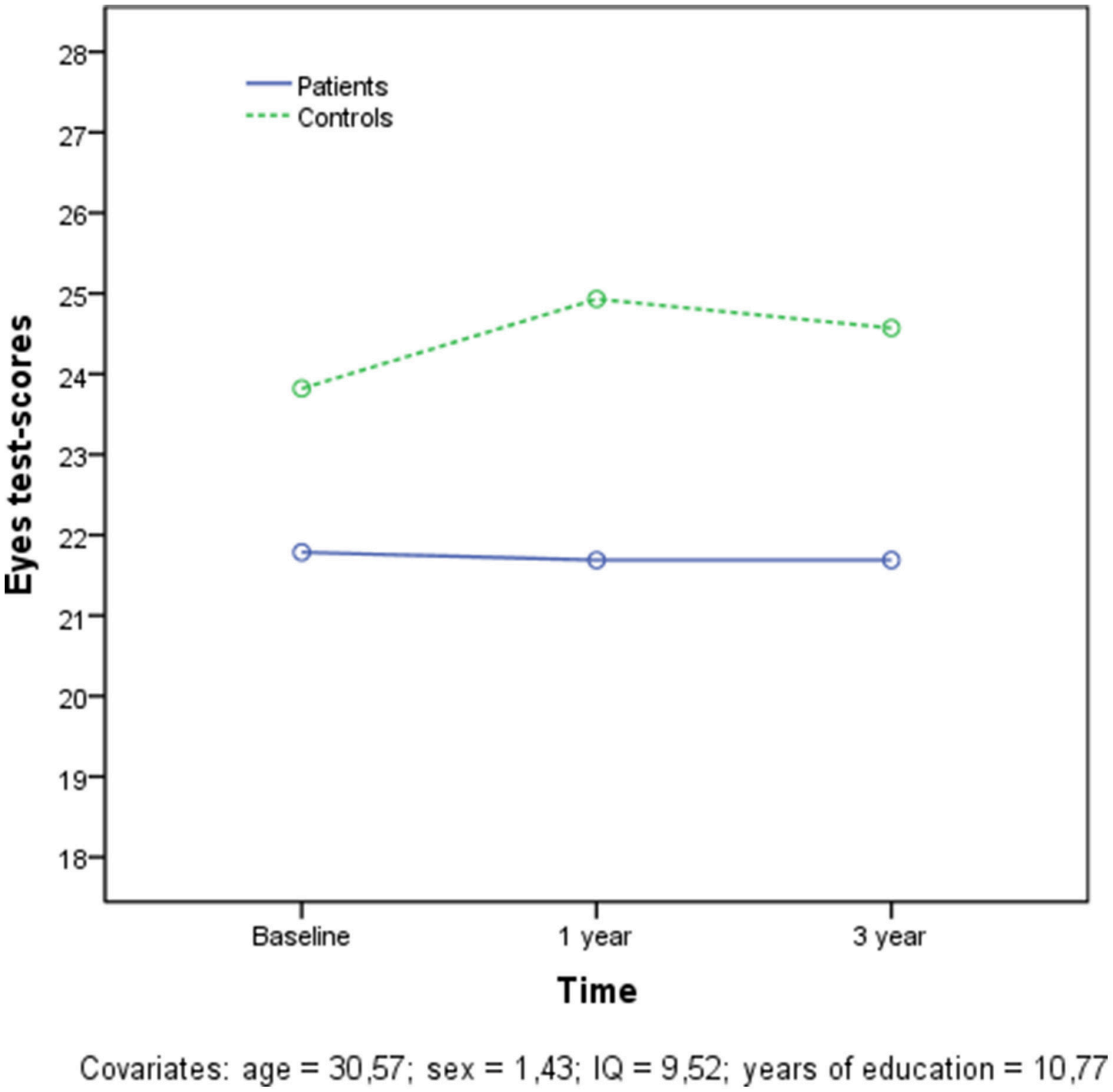
**Longitudinal results in the Eyes test for both patients and controls**.

Between-group effect on Eyes test for 107 patients and 111 healthy controls at baseline, 1-year and 3-years follow-up assessments revealed a significant main effect of group (*F* = 31; *df* = 1; *p* < 0.001), showing the patients significant impairment. There were no significant effects in within-group and time-by-group interaction in the Eyes test performance. Age (*F* = 4.04; *df* = 1; *p* < 0.046), education (*F* = 4.16; *df* = 1; *p* < 0.042) and particularly premorbid IQ (*F* = 28.2; *df* = 1; *p* < 0.001) were significant covariates (see Figure 2).

### Relationships of ToM with clinical and neurocognitive variables

We conducted correlations analyses among clinical and neurocognitive variables presented in Table 2. Due to the large number of variables analyzed, Bonferroni correction was applied to the level 0.003 (0.05/15). After Bonferroni correction, the Eyes test did not correlate with any baseline or follow-up clinical variables: however, it moderately correlated with all baseline and 1 and 3-year follow-up neuropsychological measures (all *p* < 0.003, except verbal memory).

**Table 2:** **Correlations of Eyes test with baseline, 1-year and 3-year clinical and neurocognitive variables**.

	***n***	**Baseline**	**1-year**	**3-year**
**CLINICAL VARIABLES**				
Age at onset	152	0.103		
DUP	152	−0.025		
SAPS	(152/139/132)	−0.039	−0.041	−0.069
SANS	(152/138/131)	−0.148	−0.201^*^	−0.117
Psychotic dimension	(152/139/132)	−0.016	−0.034	−0.06
Negative dimension	(152/138/131)	−0.101	−0.178^*^	−0.097
Disorganized dimension	(152/140/132)	−0.037	−0.052	−0.063
CDDS	(152/141/133)	0.06	−0.126	0.205^*^
**NEUROCOGNITIVE VARIABLES**				
Verbal memory	(152/128/118)	0.329^**^	0.176^*^	0.194^*^
Visual memory	(152/127/118)	0.277^**^	0.276^**^	0.284^**^
Working memory	(152/126/118)	0.273^**^	0.321^**^	0.345^**^
Executive function	(145/118/113)	0.241^**^	0.297^**^	0.262^**^
Processing speed	(152/128/118)	0.28^**^	0.351^**^	0.357^**^
Motor dexterity	(152/128/118)	0.351^**^	0.381^**^	0.419^**^
Attention	(150/125/117)	0.47^**^	0.29^**^	0.404^**^

SAPS, Scale for the Assessment of Positive Symptoms; SANS, Scale for the Assessment of Negative Symptoms; CDSS, Calgary Depression Scale for Schizophrenia.

* p < 0.05.

** p < 0.003.

**Figure 3:**
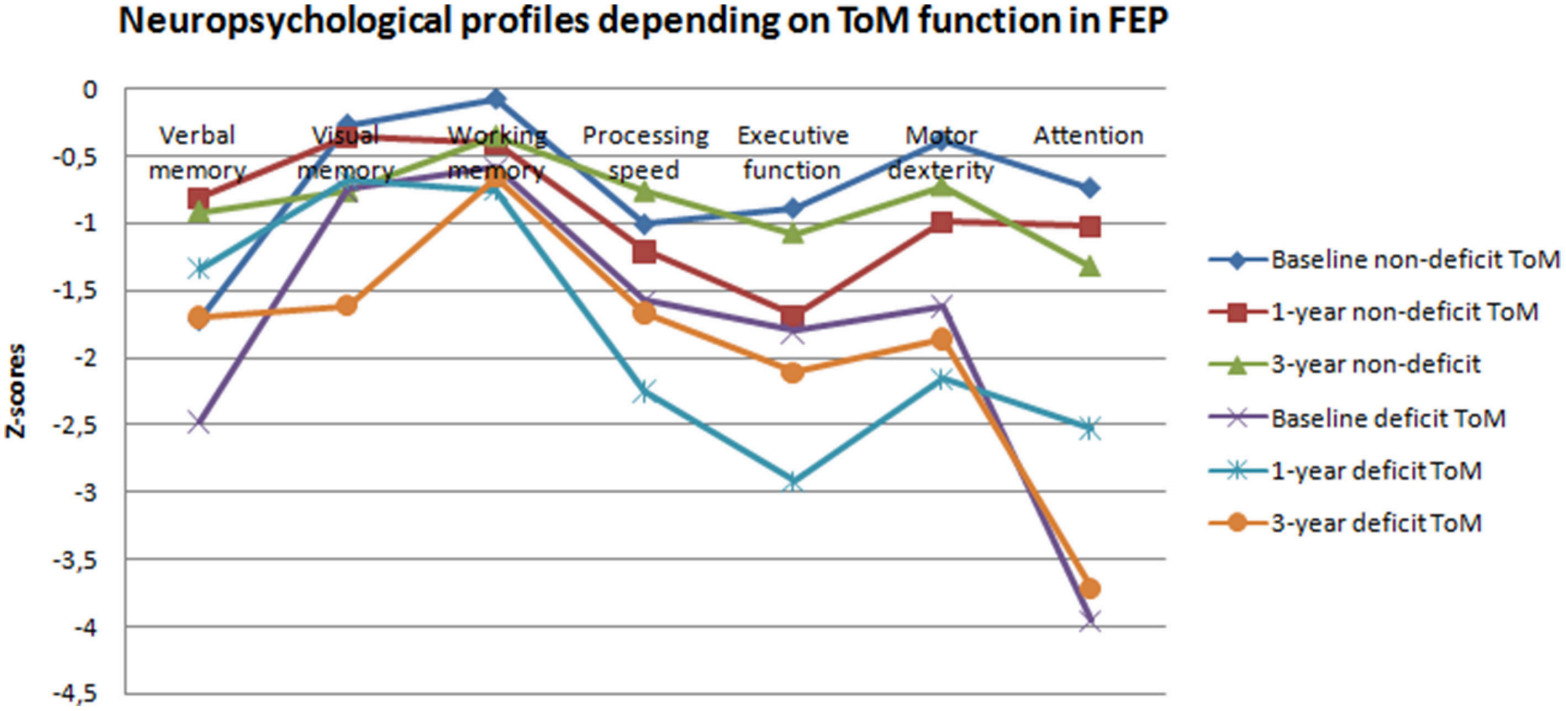
**Neuropsychological profiles according on ToM function in first episode psychosis patients**.

In order to further explore the relationship between ToM and neurocognive function we subgrouped patients according the Eyes test's mean score in healthy subjects (Eyes test ≤ 24 = deficit ToM; Eyes test > 24 = normal ToM). Based on those criteria, 119 (78%) patients presented low performance vs. 33 (22%) which were classified as normal at Eyes test baseline assessment. Using the same methodology, at 1 and 3-year follow-up, 34 (26%) and 40 (33%) exhibited efficient ToM vs. 98 (74%) and 83 (67%) showing Eyes test underperformance, respectively. The deficit and non-deficit ToM subgroups showed significant differences in all seven cognitive domain scores at baseline (all *p* < 0.017), all but visual memory and executive functions at 1-year follow-up, and all but working memory and executive functions at 3-year follow-up. Z-scores comparisons of neuropsychological performance in the three assessment moments are presented in Table 3 and the neuropsyhological profiles in each ToM subgroup and assessment time can be consulted in Figure 3.

**Table 3:** **Paired *t*-test comparisons of Z-scores neuropsychological performance in ToM subgroups at baseline, 1 and 3-year follow up**.

**ToM**	**Baseline**		***p***	**1-year**		***p***	**3-year**		***p***
	**Non-deficit**	**Deficit**		**Non-deficit**	**Deficit**		**Non-deficit**	**Deficit**	
***n***	**33 (22%)**	**119 (78%)**		**34 (26%)**	**98 (74%)**		**40 (33%)**	**83 (67%)**	
Verbal memory	−1.72	−2.48	0.004	−0.81	−1.34	0.027	−0.91	−1.7	0.002
Visual memory	−0.27	−0.76	0.015	−0.36	−0.68	0.107	−0.76	−1.61	0.001
Working memory	−0.08	−0.59	0.013	−0.41	−0.76	0.021	−0.35	−0.66	0.074
Processing speed	−1.01	−1.58	0.011	−1.21	−2.25	0.001	−0.76	−1.67	< 0.001
Executive function	−0.89	−1.81	0.016	−1.69	−2.92	0.111	−1.07	−2.11	0.12
Motor dexterity	−0.39	−1.62	< 0.001	−0.99	−2.16	0.005	−0.72	−1.86	< 0.001
Attention	−0.74	−3.96	< 0.001	−1.03	−2.53	0.03	−1.32	−3.71	0.003

### Regression model of the relationship between neurocognitive performance and ToM

Logistic regression analyses were conducted to determine the independent effect of the seven cognitive domains on ToM. Table 4 lists the results obtained. Processing speed (OR = 1.635; 95%CI 1.049–2.55; *p* = 0.03) was the unique significant contributor to efficient ToM, showing a trend toward significance in visual memory (OR = 1.431; 95%CI 0.949–2.158; *p* = 0.087). These regressions showed a well fitted model [$\chi_{\left(7\right)}^{2}$ = 24.047, *p* = 0.001, R^2^ Nagelkerke = 0.255] that predicted ToM performance with 73.9% accuracy and classified correctly 87.8% of patients with efficient ToM and 45.9% of patients with ToM impairment.

**Table 4:** **Neurocognitive predictors of ToM in FEP at 3-year follow-up**.

	***B***	***SE***	**Wald**	**Significance**	**OR**	**95% CI**	
**PREDICTORS (*n* = 118)**							
Verbal memory	0.181	0.180	1.018	0.313	1.199	0.843	1.705
Visual memory	0.358	0.210	2.921	0.087	1.431	0.949	2.158
Working memory	−0.204	0.280	0.530	0.467	0.816	0.471	1.412
Executive function	−0.041	0.085	0.231	0.631	0.960	0.813	1.134
Processing speed	0.492	0.227	4.708	0.030	1.635	1.049	2.550
Motor dexterity	0.196	0.185	1.118	0.290	1.216	0.846	1.748
Attention	0.038	0.059	0.404	0.525	1.038	0.924	1.167

Model $\chi_{\text{(7)}}^{\text{2}}$ = 24.047, p = 0.001, R^2^ Nagelkerke = 0.255.

## Discussion

This study investigated ToM deficits in FEP patients while overcoming previous studies' limitations. We confirmed that impairment in ToM is a stable trait feature in the largest sample of FEP patients analyzed to date with a longitudinal 3-year follow-up design.

Research in this field suggests that many FEP patients experience a range of difficulties in recognizing the emotions and intentions of others, as well as reflecting upon and questioning their own thinking. However, it was unclear the extent to which these deficits are stable over time, and their associations with core aspects of the disorder, such as symptoms and neurocognition. Our results showed that ToM deficit in FEP are present at illness onset, remains long-term stable, and are not related with clinical symptoms but neuropsychological deficits. Results support the notion that ToM dysfunction may be characteristic of psychosis, which does not change with treatment, consistent with the assertion that deficits in ToM are a trait of schizophrenia (Lysaker et al., 2011). The persistence of ToM deficits, even in remitted patients, suggests that they are trait related metalizing impairments (Bora et al., 2008). Previous studies found metacognitive deficits even in patients who were totally free of residual symptoms (Hamm et al., 2012), whereas some studies suggest ToM abilities seem to be related to both, positive and negative symptoms (Kohler et al., 2010). We did not find association between ToM and with clinical symptoms, neither in the acute nor in the stable phase.

Concerning neurocognitive results, we interpreted the ToM deficits as being related to a more general cognitive dysfunction confirmed in schizophrenia and FEP patients (Bora et al., 2006, 2008). Multiple cognitive deficits could mediate the observed impairments of ToM ability (Bora et al., 2009). In the present study, inefficient processing speed emerges as the neurocognitive domain specifically related to ToM dysfunction. Processing speed and episodic memory are the domains most frequently reported as significantly impaired in a large body of schizophrenia studies (Schaefer et al., 2013).

Two theoretical approaches have explained ToM deficits in autism spectrum disorders (Wilkinson and Ball, 2012). The “Theory-Theory” hypothesizes that the inference of others' mental states is an innate and learner orientated specific domain, suggesting a developmental trajectory for the understanding of mental state concepts. This approach, notably supported by Baron-Cohen, argues that there are selective deficits in ToM develop over time, resulting in individuals inability to integrate social information (e.g., attentional cues) and make effective mental state inferences about other individuals. On the contrary, “Simulation-Theory” proposes that inferences about others' mental states arise by imagining oneself in the position of the other person and simulating what they might believe, desire or intend. The argument is that the difficulty experienced by individuals in understanding mental states reflects a deficit in some aspect of the simulation process, feeding incorrect information into the simulation procedure (i.e., information relating mainly to their own perspective). A new position, called “hybrid consensus,” considers the interactions of both theories and their interdependencies, and may represent the future for empirical evidence. The indications are that pervasive domain general deficits in, for example, executive functioning and working memory, may provide some of the key components for an explanation of the wide range of developmental difficulties seen in autism and schizophrenia spectrum disorders.

It is reasonable that poor emotion recognition, found in patients with schizophrenia spectrum disorders, may be more than just impaired in the capacity to correctly infer emotions from the eyes of other people. Patients may struggle to understand other's emotions when they also are less able to process and integrate information coming from many channels into complex ideas about the self and others (Lysaker et al., 2014). Our results are in accordance with McGlade et al. (2008), confirming that mental estate decoding is mediated by global cognitive function, and particularly by the influence of processing speed. Rehabilitation of sensory processing, in both its speed and its accuracy, could improve ToM ability.

Although previous studies have not found significant effect of age on performance in ToM tasks (Green et al., 2012), our results confirm age as a moderator on the Eyes test. The older we are, the more accurate our recognition of facial emotion. The potential moderating influences of IQ deficits on ToM performance in remitted patients, as well as the potential interactions involving education have been previously suggested (Kettle et al., 2008; Bora et al., 2009). Regretfully, the problem of how IQ interferes with ToM performance remains unresolved, in part because the IQ and ToM test used in the available studies vary considerably (Brüne, 2005a). With respect to years of education, we found this variable a moderator in Eyes test performance. Using a similarly recruited Spanish sample population, with a total of 358 participants from both sexes and an age range from 18 to 65, the Fernández-Abascal et al. (2013) study, conducted a test re-test reliability of the Eyes test. All participants attended tertiary education, following our criteria of a mean of 14 years of education and thus were matched on years of education (10.38 and 10.76 years respectively). Eyes test mean scores in Fernandez-Abascal (27.18, SD 3.59) differed nearly by 4 points from our healthy control group (23.77, SD 4.31). We suggest differences in education could explain differences in Eyes test scores in these two Spanish healthy sample populations. This could be consistent with our results that found education as a moderator of Eyes test performance. Such a finding, while deserving further investigation, is encouraging when considering the use of training programs to improve social cognition (Rocha and Queirós, 2013; Sacks et al., 2013). This is of particular note because it supports the notion that education could improve social cognition.

The main strengths of this study lie in its sampling and design. We used two large and homogeneous samples, representatives of FEP patients and healthy control subjects, from the same catchment area; the 3-year longitudinal design and the employment of an instrument for ToM assessment with proven good psychometric properties (see systematic review Vellante et al., 2013). However, the study has several limitations. First, a data analytic consideration was the presence of missing data. Of the 160 FEP patients and 159 healthy controls initially included, 111 in each group entered the 3-year ANCOVA model. Second, the effect of medication was not examined. Patients were taking antipsychotic medications that might have influenced performance. It should be said though that previous studies have not found evidence for a medication effect on ToM performance because deficits in emotion perception were found at illness onset and show minimal response to effective antipsychotic treatment (Herbener et al., 2005; Chen et al., 2012). Third, in accordance with Kettle et al. (2008), deficits could also reflect problems in semantic knowledge of mental state terms in the Eyes test in the FEP group. Although all participants were provided a glossary of mental state terms used in the test, our data found relationship between Eyes test performance and WAIS-III vocabulary subtest. Finally, we did not analyze the relationship between ToM and real world functioning due to it being beyond the scope of the present paper.

In sum, the findings of this study confirmed stable, thus trait ToM deficit in FEP patients. The relationship between ToM deficits and neurocognitive function, as well as the possible link with daily functioning, support the implementation of training programs for social cognitive impairments in FEP patients.

## Author contributions

All the authors have participated and have made substantial contributions to this paper. RA: design, statistical analysis, interpretations of data and drafting the article. ES, MF, and MR: statistical analysis, interpretations of data and revising the article. KN and AF: interpretations of data and revising the article. SO, JR, and BC: conception, design and revising.

## Funding

This work was supported by the Instituto de Salud Carlos III (FIS CP07/00008 and PI14/00918) and Fundación Instituto de Investigación Marqués de Valdecilla. No pharmaceutical industry has participated in the study.

### Conflict of interest statement

The authors declare that the research was conducted in the absence of any commercial or financial relationships that could be construed as a potential conflict of interest.

## Abbreviations

FEP: First Episode Psychosis
ToM: theory of mind.
